# Nasty Notices

**Published:** 1847-03

**Authors:** 


*Nasty Notices.*—A foreigner looking over the English newspapers must think the people for whom they are printed, the nastiest on the face of the earth. No language, however gross, indelicate, offensive, or disgusting, is considered unsuited to the eyes polite which scan the contents of the "usual channels of information." At the head of the columns appears the "doctor's" book-advertisement on costiveness, with all its evils embellished with words and expressions suited to the subject, and to it alone; then the instrument-maker's flourish of syringes, operative vases, fountains, and other delicate contrivances; and at the end, the plumber's announcement of water-closets, stationary and portable. This is no fanciful picture. We see it every day. But the abomination is not consummated until the fistula-curer closes up the march, and here he is with the Lord Mayor and all his merry men in his train:—

"*Infirmary for Fistula.*—A deputation from the Fistula Infirmary, consisting of Mr. John Masterman, M. P., treasurer, the Rev. Dr. Vivian, chairman of the committee of management, Mr. Frederick Salmon, honorary surgeon, and Mr. William Carter, the secretary of the charity, waited yesterday on the right Hon. the Lord Mayor, to request his lordship's acceptance of the presidency of the infirmary for the ensuing year. The deputation was received by his lordship with the greatest courtesy, who expressed his desire to further the interests of the institution in every possible way, and fixed Monday, April the 19ht, for the anniversary festival of the institution, at which he signified his intention to preside."

The *quid nunc* family of Cockneyland is at present all in an uproar of most amiable indignation against the horrid Irish, who will not starve quietly; and words can scarcely be found sufficiently strong to express the contempt entertained for Hibernian delinquencies; but we challenge the greatest advocate of Saxon refinement to match the above paragraph in Celtic barbarity. Just let us picture to ourselves the Lord Mayor of Dublin seated in state to receive a deputation headed by one of the city members ushering in a fistula-curer to bespeak his lordship's patronage and custom; and soliciting the first municipal officer of the city to "accept the presidency" of his filthy "institution!" Such a thing could not take place amongst us, or if it did, we should not at all events have it thus blazoned forth.—***Dub. Med. Press.***

